# Androgen receptor affects the response to immune checkpoint therapy by suppressing PD-L1 in hepatocellular carcinoma

**DOI:** 10.18632/aging.103231

**Published:** 2020-06-24

**Authors:** Guangyi Jiang, Liang Shi, Xueyong Zheng, Xinjie Zhang, Ke Wu, Boqiang Liu, Peijian Yan, Xiao Liang, Tunan Yu, Yifan Wang, Xiujun Cai

**Affiliations:** 1 Department of General Surgery, Sir Run Run Shaw Hospital, College of Medicine, Zhejiang University, Hangzhou, Zhejiang, China; 2 Key Laboratory of Laparoscopic Technology of Zhejiang Province, Sir Run Run Shaw Hospital, College of Medicine, Zhejiang University, Hangzhou, Zhejiang, China

**Keywords:** immune surveillance, tumor microenvironment, PD-1/PD-L1 pathway, androgen receptor

## Abstract

Hepatocellular carcinoma (HCC) is a heterogeneous malignancy with gender-related differences in onset and course. Androgen receptor (AR), a male hormone receptor, is critical in the initiation and progression of HCC. The role of AR in HCC has been mechanistically characterized and anti-AR therapies have been developed, showing limited efficacy. Immunotherapy targeting immune checkpoint proteins may substantially improve the clinical management of HCC. The mechanism by which AR influences HCC immune state remains unclear. In this study, we demonstrated that AR negatively regulated PD-L1, by acting as a transcriptional repressor of PD-L1. Notably, AR over-expression in HCC cells enhanced CD8^+^T function *in vitro*. We then verified the AR/PD-L1 correlation in patients. In animal experiment we found that lower AR expressed tumor achieved better response to PD-L1 inhibitor. Thus, AR suppressed PD-L1 expression, possibly contributing to gender disparity in HCC. Better understanding of the roles of AR during HCC initiation and progression will provide a novel angle to develop potential HCC immunotherapies.

## INTRODUCTION

Hepatocellular carcinoma (HCC) is the sixth most common malignant tumor in the world. The mortality of primary liver cancer has dropped from the second to the fourth highest in the past five years, but the incidence is increasing in both developing and developed countries [1]. Many pathogenic factors can lead to the development of liver cancer, such as viral hepatitis, cirrhosis, exposure to cancerogenic substances, and other chronic liver damages [2–5]. Moreover, HCC is characterized by gender disparity, with a 2:1 to 7:1 male-to-female ratio in disease incidence [6].

Androgen receptor (AR) is a highly expressed male hormone receptor which, upon stimulation by androgens such as testosterone, translocate into the nucleus, where it binds to androgen response elements (AREs) and regulates downstream genes [7]. AR was first identified as a tumor-promoting gene in prostate cancer. Since AR is implicated in all stages of prostate cancer, AR antagonists have been developed for AR-targeted therapy [8–10]. In 2012, an AR inhibitor was approved by FDA for prostate cancer and yielded promising results [11]. In line with the observed gender disparity of the disease, AR contributes to the initiation and progression of HCC [12–14]. However, the molecular details of these effects have not been elucidated. Nonetheless, AR was identified as a therapeutic target for HCC [13]. Unfortunately, the results of early clinical trials testing anti-androgen therapies in liver cancer were disappointing [15, 16].

During the last decades, liver cancer treatment has become a global hotspot and researches effort toward prevention and treatment were mainly focused on liver cancer cells [17, 18]. In recent years, the role of tumor microenvironment in the occurrence and development of liver cancer has been widely recognized [19, 20]. Programmed death ligand-1 (PD-L1), a microenvironment transmembrane protein, is an immune checkpoint factor participating in immune surveillance and exerting suppressive effects on the immune system [21–23]. Inhibition of the PD-1/PD-L1 pathway has proven effective in many types of cancer, including HCC.

Many recent studies have focused on HCC diagnosis and immune therapy. In particular, the study of Hu K et al [24] demonstrated that the PD-L1/CLEC1B combination associates with poor outcome in HCC patients. Another study proved the safety and effectiveness of the PD-L1 inhibitor, nivolumab, in the treatment of patients with advanced HCC [25]. The US Food and Drug Administration recently approved nivolumab for the treatment of sorafenib-refractory advanced HCC. The reported objective response rate for nivolumab in patients with advanced HCC is 20%, therefore still unsatisfactory. Notably, the correlation between gender disparity in HCC and PD-L1 has not been directly addressed.

Here, we unveiled a functional relationship between PD-L1 and AR. In particular, AR was found to directly bind to the PD-L1 promoter, downregulating its expression and execute negative effects on anti-PD-L1 immunotherapy *in vivo*.

## RESULTS

### Androgen receptor suppresses PD-L1 expression in HCC cells

**Figure 1:**
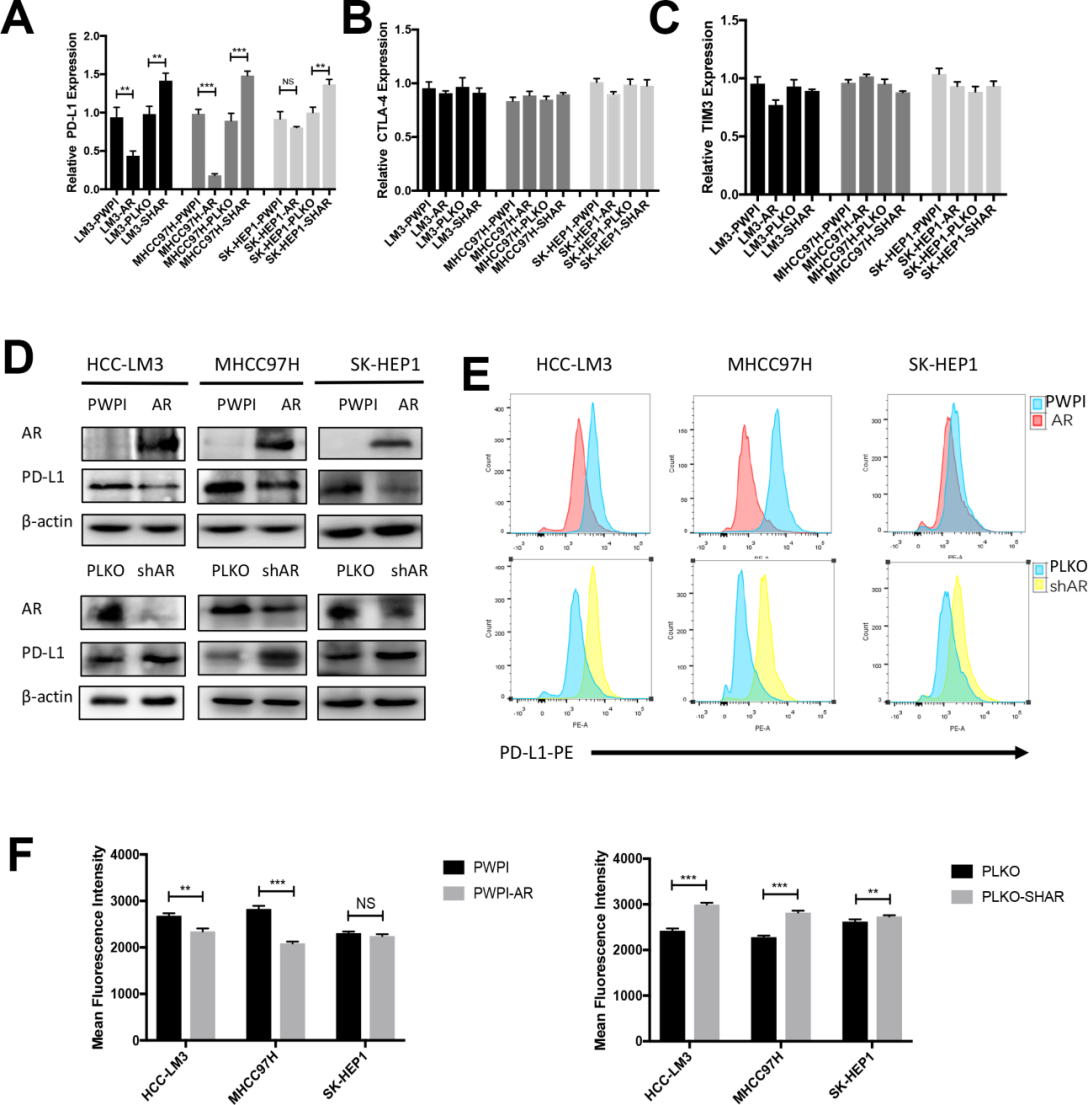
**Modulation of AR influence the expression of PD-L1 in HCC cells.** (**A**–**C**) RT-qPCR analysis of three checkpoints in over-expression AR and down-expression AR HCC cells. (**D**) Western Blot of AR and PD-L1 in three HCC cell lines. (**C**) Flow cytometry of the membrane PD-L1. (**E**, **F**) Mean fluorescence intensity of membrane PD-L1. *P<0.05, ** *P*<0.01 and *** *P*<0.001.

In an attempt to verify whether AR influenced immune checkpoint protein expression in HCC, we evaluated the impact of different AR expression levels on PD-L1 expression in various HCC cell lines. RT-qPCR showed that overexpression AR attenuated PD-L1 mRNA expression and knockdown AR increased PD-L1 mRNA expression in HCC cell lines (Figure 1A). No significant difference was observed in CTLA4 and TIM3 (Figure 1B, 1C). The analysis of western-blot consisted with RT-qPCR results (Figure 1D). Since PD-L1 is a transmembrane protein, PD-L1 membrane expression changes regulated by AR was determined by flow cytometry. We found the level of AR negatively correlated with the extent of membrane-localized PD-L1 (Figure 1E, 1F). Due to AR is a carcinogen in prostate cancer, next we tested the correlation in two prostate cancer cell line (DU145 and PC3) and no significant changes were observed (Supplementary Figure 1).

### The AR/PD-L1 axis influences the function of CD8+ T cells

**Figure 2:**
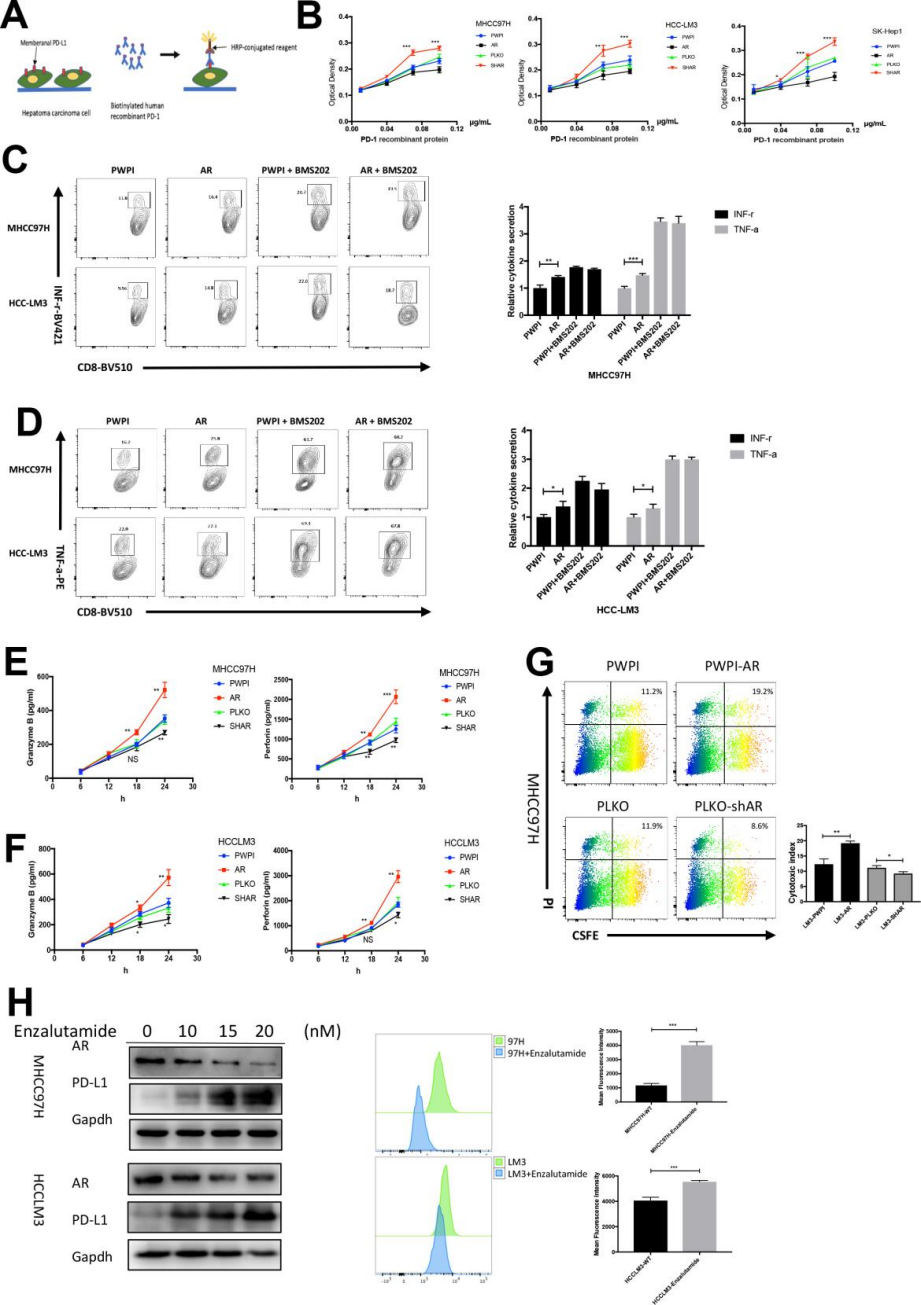
**Modulation of AR regulate the immune state.** (**A**) Schematic diagram of PD1-PD-L1 binding assay. (**B**) The results of binding assay in three HCC cell lines. (**C**) Intracellular INF-r expression in CD8^+^T cells co-cultured with HCC cells. (**D**) Intracellular TNF-a expression in CD8^+^T cells co-cultured with HCC cells. (**E**) Secreted cytokine (granzyme B and perforin) in MHCC97H cells. (**F**) ELISA of serum secreted cytokine (granzyme B and perforin) in HCCLM3 cells. (**G**) T cell cytotoxicity assay in MHCC97H with different AR expression. (**H**) AR antagonist cause change of AR and PD-L1 *in vitro*. *P<0.05, ** *P*<0.01 and *** *P*<0.001.

To verify the biological function of the HCC cell lines with different AR expression, PD-1/PD-L1 binding assay was performed. A flow diagram of the binding assay was illustrated in Figure 2A. The results identified that AR-overexpressed HCC cell lines (MHCC97H and HCCLM3) exhibited decreased PD-1 binding, while AR knockdown caused increased PD-1 binding (Figure 2B). To simulate the *in vivo* immune response, HCC cell lines with different AR expression were co-cultured with pre-activated CD8+ T cells from healthy donors, and cytokine production from CD8+ T cells was analyzed. The purity percentage of CD8+ T cells was assessed by flow cytometry (Supplementary Figure 2). We found that AR-overexpressed HCC cells stimulated more intracellular functional cytokines (INF-r and TNF-a) secreted by CD8^+^ T cells. Then we added BMS-202, a potent PD-1/PD-L1 inhibitor, into the medium and repeated the co-cultured assay. We found no significant difference was observed in intracellular cytokine secretion between AR-overexpressed and control HCC cells after blockage of the PD-1/PD-L1 pathway. (Figure 2C, 2D). The results indicated that the functional changes in T cells was caused by PD-L1.

The cytotoxicity of T cells depends on the secretion of granular enzymes and perforin. Next, we tested the serum granzyme B and perforin using ELISA. The results showed that CD8^+^ T cell secreted more granzyme B and perforin when co-cultured with AR-overexpressed HCC cells, which are key factors for the development of cytotoxicity. On the other hand, AR-downregulated HCC cells attenuated the secretion of these factors by CD8^+^ T cells (Figure 2E, 2F). To further explore the impact of AR expression on CD8^+^ T cell-mediated cytotoxicity, a 24-h cytotoxicity assay was performed in MHCC97H cells. Flow cytometry showed that AR overexpression was associated with increased tumor cell death, while MHCC97H cells with low AR expression were more likely to resist to CD8^+^ T cells (Figure 2G). To demonstrate whether AR inhibitors cause changes in PD-L1, the second-generation AR antagonist, Enzalutamide, was used. As shown in Figure 2H, Enzalutamide attenuated AR expression and increased membrane PD-L1 expression.

### AR transcriptionally represses PD-L1 via binding to its promoter

**Figure 3:**
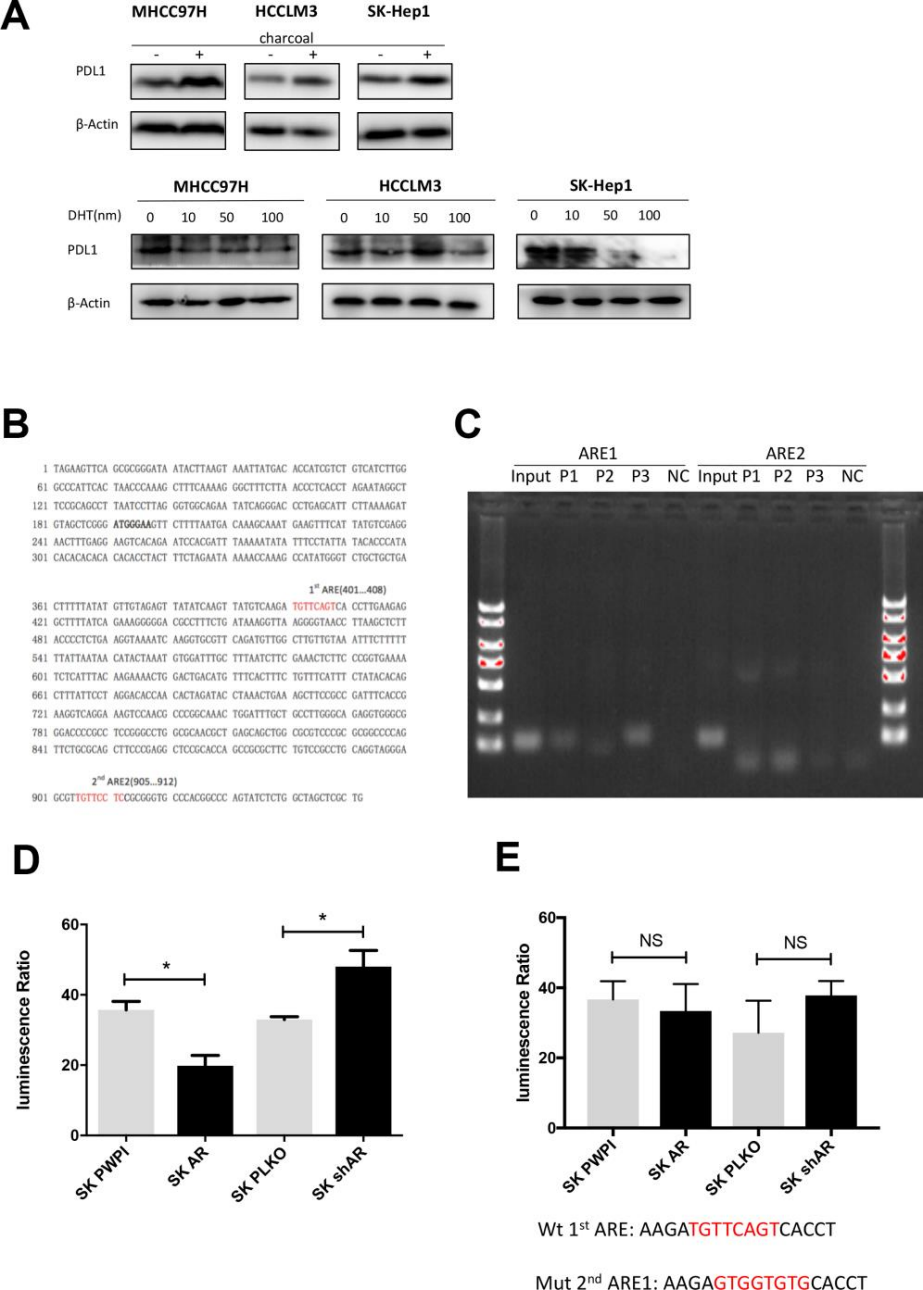
**AR activates PD-L1 transcription by binding to its promoter region.** (**A**) Castration assay was performed in three HCC cell lines. (**B**) Predicted localization of AREs in PD-L1 promoter region (red). (**C**) Chromatin immunoprecipitation was performed in wild-type SK-Hep1 cells. The detecting primer was designed based on the prediction result of potential AREs. (**D**) Wild-type PD-L1 promoter construct was transfected into SK-Hep1 cells with internal control pRL-TK. Then, we performed luciferase reporter assays with manipulated AR to detect if AR could affect activation of PD-L1 promoter. (**E**) Luciferase reporter assays were performed after transfecting mutated 1^st^ ARE into AR-overexpressed SK-Hep1 cells and AR knocked-down SK-Hep1 cells. *P<0.05, ** *P*<0.01 and *** *P*<0.001.

Androgen receptor is a transcriptional factor that functions in both androgen-dependent and androgen-independent pathway [8, 26]. In order to clarify the role of androgen in AR-mediated PD-L1 regulation, we performed an *in vitro* castration assay [27]. We found that DHT dose-dependently attenuated PD-L1 expression (Figure 3A). When the castration assay was performed in AR-negative HepG2 cells, no changes in PD-L1 expression were observed (Supplementary Figure 3). These results indicated that AR regulates PD-L1 in androgen-dependent pathway. As for the mechanism, we first speculated whether AR can be incorporated into the promoter region of PD-L1. So we analyzed the promoter region of PD-L1 (http://www.genecards.org/cgi-bin/carddisp.pl?gene=CD274) with ALGGEN-PROMO software (http://alggen.lsi.upc.es/cgi-bin/promo_v3/promo/promoinit.cgi?dirDB=TF_8.3) and identified two potential AR-responsive elements (*ARE 1* and *ARE* 2) (Figure 3B). According to this result we built a hypothesis that AR may works by combining the promoter regions of PD-L1. Next, chromatin immunoprecipitation (ChIP) assays and luciferase reporter assay were performed to verify our hypothesis using the SK-HEP1 cell line. The results demonstrated that AR could bind to ARE1 but not to ARE2 (Figure 3C). In luciferase reporter assay we found that AR had an impact on gene transcription downstream of the PD-L1 promoter (Figure 3D). Then we structured ARE1 mutation report plasmid for luciferase reporter assay and found no impact on PD-L1 promotor transcription (Figure 3E). These results demonstrated that AR suppress PD-L1 expression via binding to the PD-L1 promotor and directly attenuate PD-L1 gene transcription.

### AR is negatively correlated with PD-L1 expression in human clinical HCC samples

**Figure 4:**
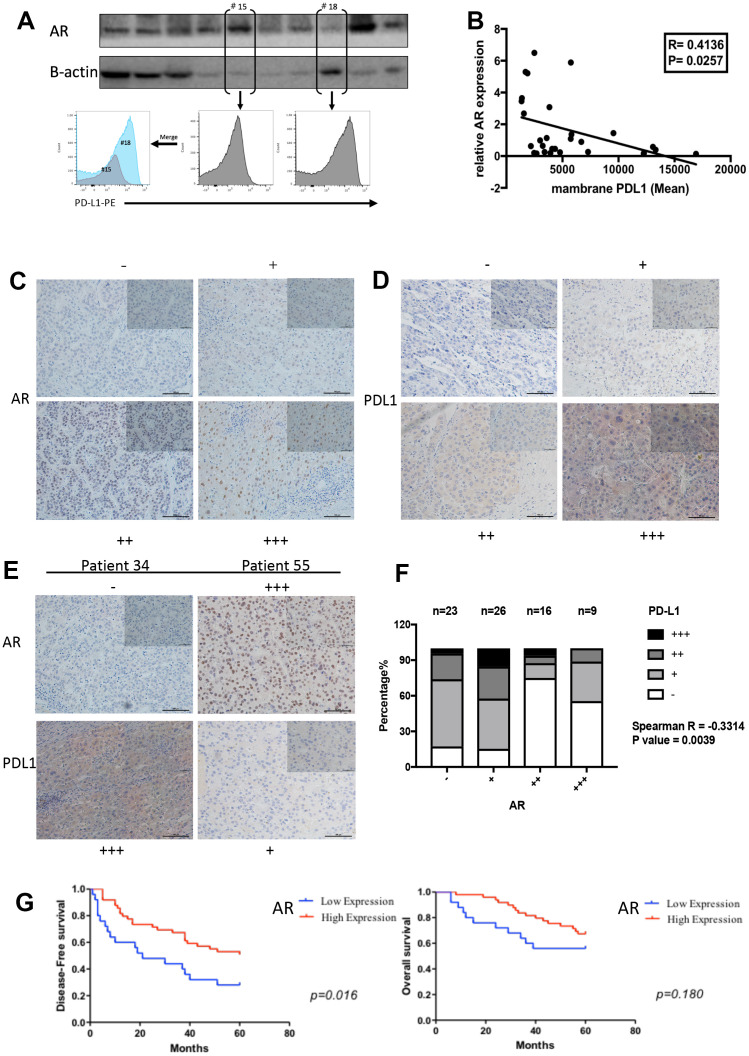
**The negative correlation between AR and PD-L1 *in vivo*.** (**A**) AR protein expression and membrane PD-L1 detection in patients’ samples (**B**) The correlation results between AR and PD-L1 (**C**) Representative images for scoring the AR IHC staining. (**D**) Representative images for scoring the PD-L1 IHC staining. (**E**) Representative images to show the comparison of AR and PD-L1 staining in the same patient. (**F**) Spearman correlation analysis for AR and PD-L1 based in our stained clinical samples (P value= 0.0039). (**G**) Survival curve analysis in different AR expression.

To verify that the results obtained with the above HCC cell lines were representative of disease-relevant events, we used GEPIA (http://gepia.cancer-pku.cn/), an interactive web server that was designed to analyze the functional relationships between genes based on published databases. By using the TCGA liver cancer database and Spearman’s test, we found a negative correlation between AR and PD-L1 expression level (Correlation coefficient = −0.20, P= 0.014) (Supplementary Figure 4). To further verify this correlation in data from our center, we collected 29 surgical samples from HCC patients from Sir Run Run Shaw Hospital for AR protein test and membrane PD-L1 detection. For standardization, AR expression was normalized to that of β-actin. Single-cell suspensions were prepared and membrane PD-L1 expression was evaluated by flow cytometry. The results showed a mild correlation between AR and membrane PD-L1 expression in patient samples (correlation coefficient = −0.413, P= 0.026; Figure 4A, 4B).

Next, AR and PD-L1 expression was analyzed by IHC in 89 samples from patients of the Sir Run Run Shaw Hospital. We classified the results into four grades (negative, weakly positive, positive, and strongly positive) according to staining intensity. Representative images are shown in Figure 4C, 4D. The immunohistochemical scores were assessed by two pathologists. The results suggested a negative correlation between the presence of AR-positive nuclei and PD-L1 expression (Figure 4E, 4F, Spearman’s R=-0.331, P=0.004). Then we performed survival analysis and the result showed that HCC patients with high AR expression experienced longer disease-free survival compared to those with poor AR expression. No significant difference was observed in overall survival between the two groups.

### AR overexpression attenuated the effects of the PD-L1 inhibitor *in vivo*

**Figure 5:**
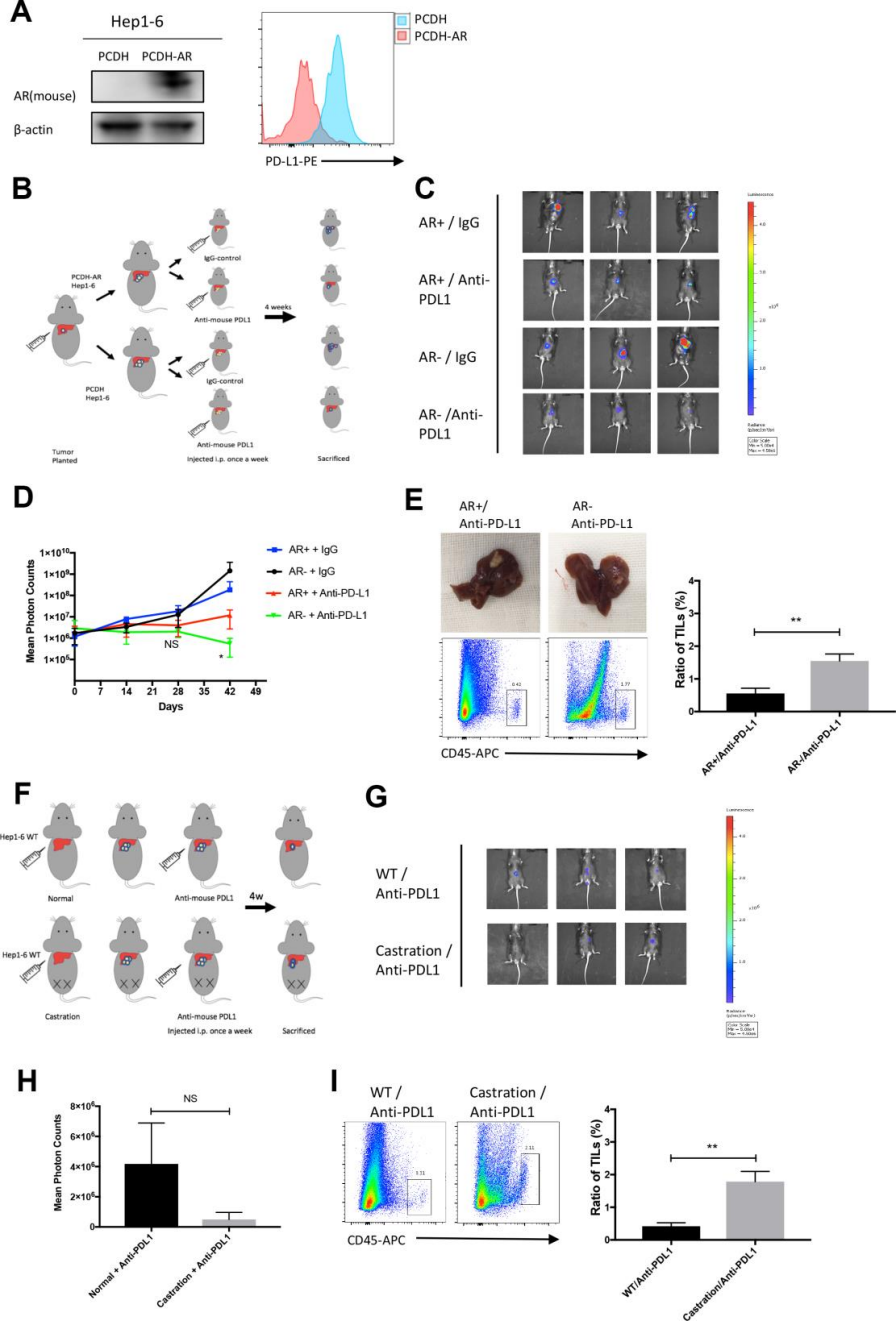
**AR overexpression attenuated the effects of the PD-L1 inhibitor *in vivo*.** (**A**) Establishment of overexpressed AR Hep1-6 and tested by western blot and flow cytometry. (**B**) Flow diagram of animal experiment. (**C**) The luminescence of tumor detected by IVIS system. (**D**) The growth curve of mice liver tumor represented by photon counts. (**E**) Picture of liver tumor and tumor infiltrating lymphocytes (TILs) detected by flow cytometry. (**F**) Flow diagram of animal experiment on castrated mice. (**G**) The luminescence of tumor detected by IVIS system. (**H**) The statistical results of the animal experiment. (**I**) Tumor infiltrating lymphocytes (TILs) detected by flow cytometry.

To verify whether AR expression had an impact on the effects of the PD-L1 inhibitor *in vivo*, we set up an orthotopic implantation model in mice using Hep1-6-PCDH(AR^-^) and Hep1-6-AR(AR^+^) cells. Anti-mouse PD-L1 antibodies or IgG2b isotype controls were i.p. injected weekly. Tumor size and range were represented by photon counts measured by using the IVIS detection system. The cell line establishment was tested by western blot and flow cytometry (Figure 5A). A flow diagram is shown in Figure 5B. IVIS images at six weeks showed that (AR^-^) tumors were smaller than (AR^+^) tumors in the group injected with PD-L1 inhibitor, while no significant differences were observed in the group with IgG2b injected (Figure 5C). The tumor growth curve was showed in Figure 5D*.* With the anti-mouse PD-L1 injection, (AR^-^) tumors gained a markedly slow growth compared with (AR^+^) tumors and no significant difference was observed in control group. We then sacrificed the mice and collected the liver lesion for further test. The ratio of tumor infiltrating lymphocytes(TILs) were verified by flow cytometry. We found that the (AR^-^) tumors treated with anti-PD-L1 had more TILs than (AR^+^) tumors (Figure 5E). Besides, we measured the testosterone level of plasma between the four groups and found no significant difference (Supplementary Figure 5A). These results indicated that AR can impact the effect of PD-L1 inhibitor and decreased the T cell infiltration.

In castration mice model, we used Hep1-6 wild type (WT) cells to establish the orthotopic implantation model and treated with anti-mouse PD-L1 weekly. The flow diagram is showed in Fig 5*F.* We first measured the testosterone level of castration group and control group to prove the successful establishment of the model (Supplementary Figure 5B). After six weeks injection, results of IVIS image detection revealed that tumors in castrated mice were smaller than tumors in normal mice (Figure 5G, 5H). The TILs were also measured and the results showed tumor in castrated mice had more lymphocytes infiltrated than that in normal mouse (Figure 5I). This result demonstrated that androgen expression also impacted the effect of PD-L1 inhibitor.

## DISCUSSION

HCC is a male-dominant tumor and AR has been verified to play a critical role in the initiation and progression of HCC, and the mortality rate of HCC is still high [28]. Lack of effective treatment is the leading cause of recurrence and death. In recent years, tumor immunotherapy has become research hotspot. PD-L1 inhibitor has been reported a promising respond in non-small cell lung cancer, melanoma, renal cell carcinoma and head and neck cancers [29], however, the response of PD-L1 inhibitor in HCC is still controversial [30]. Here, we investigated the potential impact of AR on tumor microenvironment and immune surveillance in HCC. We found that AR suppressed PD-L1 transcription, directly altered the tumor microenvironment by decreasing the membrane PD-L1 expression and enhance the function and proliferation of activated CD8+ T cells. These results may be useful in the design of a new immunotherapies strategy for HCC.

The role of androgen receptor (AR) in gender differences characterizing HCC initiation and progression has been extensively explored. However, no satisfactory treatment for HCC has as yet been developed. Although hormone therapy based on AR antagonists has been used to tackle HCC, it did not achieve the expected therapeutic effect [31]. Zhang et al*.* proposed a model that may partly explain these results, implying the activation of an AKT-mTOR-mediated feedback, which in turn would promote nuclear AR expression [32]. These authors propose a potential therapeutic strategy based on AR and mTOR co-targeting. It can partly explain the limited application of AR antagonists in treating HCC. And our study illustrates from the point of view of immunity to explain the limitation of AR antagonist.

CD274 also called PD-L1 was first reported in 2002 by Dong H et al [33]. As a novel checkpoint, tumor- associated PD-L1 plays important roles in mediating T cell activation and apoptosis, causing immune surveillance attenuated and promote tumor progression [34, 35]. Normally, PD-1 pathways and its ligands, PD-L1 and PD-L2, contribute to the balance between activation and inhibitory signals that are needed for physiologic immune system works and for the maintenance of T cell self-tolerance and homeostasis [36]. When the cells become malignancy, expression of PD-L1 increased which cause overbalance of PD-L1 and PDL-2, PD-1 which expressed predominantly by T cells physical contact and bind more ligands PD-L1, the interaction between PD-1 and PD-L1 inhibit self-reactive T cells [37]. According to the PD-1/PD-L1 binding assay in our study, the AR overexpressed HCC cell lines bind little PD-1 recombination protein than that of AR knockdown cell lines. This result demonstrated that PD-L1 changes based on AR has biological activity. Intracellular cytokine test and T cell cytotoxicity test verified that PD-L1 changes caused by AR can moderate T cell function *in vitro*.

Male gender is a risk factor for HCC [38]. The results of immunohistochemical verified the negative correlation of AR and PD-L1 in patients’ samples. Importantly, nuclear AR overexpression is positively correlated with duration of disease-free survival and no significant difference was observed in overall survival. However, according to the study of Zhang et al [14], high nuclear AR expression is significantly correlated with poorer overall survival of HCC patients. These results seem to be contradictory. Ma et al*.* [39] reported that AR-positive HCC cells exhibit a lower metastatic ability compared to AR-negative cells and hepatic-AR plays dual yet opposite roles to promote HCC progression but suppress HCC metastasis. This result could reasonably explain our findings. As for the overall survival period, insufficient sample size and the follow-up deadline of five years may cause the result.

Tumor infiltrating lymphocytes (TILs) are key players in antitumor response. The function of the infiltrating lymphocytes is significantly affected by the tumor microenvironment [40]. One of the mechanisms is lacking of co-stimulatory molecules (such as CD80 and CD86), which is caused by the tumor provided inflammatory background and lead TILs become “exhausted” [41, 42]. Another mechanism is the existence of the co-inhibitory signal pathway such as PD-L1 and CTLA-4, the blockage of the inhibitory receptors can restore and ever enhance the function of the TILs [32]. In our study, we firstly identified that AR can regulate the expression of PD-L1 with no CTLA-4 changes. According to the result of animal experiment, AR negative tumor gained more T cell infiltrated than AR positive tumor when treated with PD-L1 inhibitor. This result verified that the expression of AR can impact the response of PD-L1 inhibitor. We also detected more T cell infiltrating in castrated mice than that in normal mice. The result demonstrated that the role of AR/PD-L1 pathway requires androgen involvement.

Interestingly, only half of the predicted canonical AREs were confirmed by ChIP analysis of the PD-L1 promoter. Normally, AR binds to ARE as a homo-dimer. However, one report demonstrated that AR bound to half-site-like sequences could still be transcriptionally functional [43], which may support our current findings.

Moreover, AR is known to be crucial for the development of prostate cancer [44, 8], but we did not detect any correlation between AR and PD-L1 in prostate cancer. Thus, the negative correlation between these factors may be a specific trait of hepatocellular carcinoma.

## CONCLUSIONS

In summary, our findings indicated a novel function of androgen receptor, which transcriptionally suppressed PD-L1 in HCC cells by direct binding to the relevant promoter. Different AR expression in HCC cells can cause changes in the immune response. Our finding provided new hints for the development of a new immunotherapeutic approach for hepatocellular carcinoma.

## MATERIALS AND METHODS

### Cell culture and transfection

Human HCC cells were maintained in DMEM (Invitrogen, Grand Island, NY) with 10% fetal bovine serum (FBS), 1% glutamine, and 1% penicillin/ streptomycin. After cells were used within 3 months of resuscitation. Hep1-6 mouse liver cancer cells were kindly provided by the School of Basic Medicine, Zhejiang University. PC3 and DU145 prostate cancer cells were obtained from the Laboratory of Urinary Surgery, Sir Run Run Shaw Hospital. Cell lines were cultured in a 5% (v/v) CO2 humidified incubator at 37 °C. HCCLM3, MHCC97H, and SK-Hep1 were obtained from ATCC (Manassas, VA, USA) and authenticated by a professional biotechnology company. For androgen free media preparation, FBS was pretreated using dextran-coated charcoal (C6241, Sigma, Shanghai, China) overnight. Then, the charcoal was removed by filtration and charcoaled FBS was collected for further experiments.

And the stable transfectants were established from HCC cell lines, as previously described [45]. Briefly, HEK-293T cells were transfected using the core plasmid (pWPI, pWPI-AR, pLKO1, pLKO1-shAR) with the psAX2 packaging plasmid and pMD2G envelope plasmid, and then incubated for 48 h to obtain lentivirus supernatant, which was frozen at −80°C for further cell infection and production of stable clones. We established 4 stable transfection cell types of each cell line called pWPI, pWPI-AR, pLKO, pLKO-shAR.

### T cell separation and activation

The whole blood was obtained from three health donors. Gently added the whole blood in cell separation (Lympholyte CL5020) media at a ratio of 1:9 and balancing. Then centrifuge in the condition of 400G for 35 minutes at room temperature with a rising speed of 9 and a falling speed of 1. After centrifugation, the white suspension of the intermediate layer is slowly sucked out, which is the peripheral blood mononuclear cells(PBMC). Then CD8^+^T cells were purified using MojoSort™ Human CD8 Nanobeads (Biolegend 480107) and maintained in 1640 medium (Invitrogen) with 30 U/ml IL-2 (Pepretech 20020).

Purified CD8^+^ T cells were activated using Dynabeads™ Human T-Activator CD3/CD28 for T Cell Expansion and Activation (Thermo Fisher 11161D). Briefly, resuspended Dynabeads were added into culture medium at a bead-to-cell ratio of 1:1 and incubation for 4 days in a humidified CO2 incubator at 37°C. Then the activated T cell was collected for further experiments.

### Intracellular cytokine detection and flow cytometry

The HCC cells were co-cultured with activated T cells for 48h at the ratio of 1:1. Brefeldin A (BD Biosciences) was pretreated with the mixed cells for 6 h. After permeabilizing using fixation and permeabilization reagents (Biolegend 426803), the cells were stained with anti-human CD8-BV510 antibodies (Biolegend 344731), anti-human INF-r-BV421 (Biolegend 506537) and anti-human TNF-a-PE (Biolegend 502908) for 20 mins. Then the samples were ready for flow cytometry.

To analyze membrane PD-L1, HCC cells were dissociated with trypsin-EDTA solution and stained with anti-human CD274 PE antibodies (Biolegend 329705) and incubated for 20 min. Then the cells were harvested for flow analysis.

For PD-L1 flow cytometry from patient samples, we prepared a single-cell suspension from patient samples using MagicFilter and MagicVajra (Bozhentech B160103). Then the suspended cells were stained with APC anti-human CD45 antibodies (Biolegend 304012), Brilliant Violet 510 anti-human CD8 antibodies (Biolegend 344732), FITC anti-human CD3 antibodies (Biolegend 300406), PE anti-human CD274 antibodies (Biolegend 329706), and 7-AAD Viability Staining (Biolegend 420404). After a 20 mins staining, cells were re-suspended in 300 μL of PBS for flow analysis.

### PD-1-PD-L1 binding assay

To test the binding of membrane PD-L1 from HCC cells to PD-1, HCCLM3 cells were seeded in 96-well plates and incubated overnight. Then, concentrations gradient of biotin-labeled human PD-1 protein (Acro Biosystem H82F3) were added, and incubation performed for 2 h at 37 °C. Next, 100 μL of horseradish peroxidase-conjugated streptavidin (BD Bioscience) diluted in PBS containing 0.1% BSA were added in each well, followed by incubation for 1 h at 37 °C. The color reactions were developed with tetramethylbenzidine (Pierce) and stopped using 0.5 N H_2_SO4. Finally, the absorbance at 450 nm was measured with a BioTek plate reader.

### ELISA

The co-cultured medium was collected for detection of Granzyme B and Perforin using ELISA kits (Biolegend 439207and Thermofisher BMS2306). The ELISA kit used for plasma testosterone level measure was purchased from Abcam(ab108666). The standard curve was made to determine the concentration. All the procedures were performed according to the manufacturer's instructions.

### Quantitative real-time PCR analysis

The total RNAs were isolated using Trizol(Invitrogen). One microgram of total RNA was subjected to reverse transcription using Superscript III transcriptase (Invitrogen). Quantitative real-time PCR (qRT-PCR) was performed using Bio-Rad CFX96 system (Bio-Rad, Hercules, CA) with SYBR green to determine the mRNA expression level of the target genes. Expression levels were normalized to the expression of GAPDH mRNA (see Supplementary Table 1 for details).

### Western blot analysis

The cells or tissues were lysed in RIPA buffer and proteins were separated on 8–10% SDS/PAGE gels and transferred onto PVDF membranes (Millipore, Billerica, MA). After blocking using 5% BSA, the membranes were incubated overnight with the appropriate dilutions of specific antibodies, then the blots were incubated with HRP-conjugated secondary antibodies and detected using an ECL system (Thermo Fisher Scientific, Rochester, NY, USA). Anti-GAPDH (1:1000, 6c5) and anti-AR (1:1000, N20) antibodies were purchased from Santa Cruz Biotechnology (Santa Cruz, CA). The anti PD-L1 antibody (Cell Signaling Technology 13684) was purchased from Cell Signaling Technology.

### Castration assay

HCC cells were cultured in charcoaled medium (all steroid hormones were removed by charcoal adsorption) for 48 h, and then treated with gradient dihydrotestosterone (DHT) for another 48h. Then protein was extracted and Western Blot was performed.

### Plasmid construction and luciferase assay

The AR shRNA was inserted into pLKO1 vector and transfer to 293T for lentivirus generation. The AR overexpression plasmid was provided from Laboratory of Urinary Surgery, Sir Run Run Shaw Hospital and inserted into PWPI vector using Gibson assembly method. The mouse AR overexpression plasmid (Qingke, China) was inserted into PCDH vector using Gibson assembly method. For the luciferase reporter assay, the full-length promoter of PD-L1 was obtained from genomic DNA of 293T cells by Phusion® High-Fidelity DNA Polymerase (NEB, Beverly, NY) and conjugated into a pGL3-basic vector (Promega, Madison, WI). For the ARE mutation, quick change was used according to the production’s instruction.

For the luciferase assay, cells were plated in 24-well plates and Lipofectamine3000 (Invitrogen) was used for cDNA transfection, according to the manufacturer’s instructions. The plasmid pRL-TK was used as an internal control. Luciferase activity was detected by a Dual-Luciferase Assay system (Promega).

### Chromatin immunoprecipitation assay (ChIP)

Cell lysates were sequentially pre-cleared with normal rabbit IgG (sc-2027, Santa Cruz Biotechnology) and protein A-agarose (Santa Cruz Biotechnology). The anti-AR antibody was purchased from Santa Cruz (2.0 μg) and added to the cell lysates and incubated at 4 °C overnight. Input was used as positive control and IgG was used as negative control. The specific primers were designed to amplify the target sequence within the human PD-L1 promoter and listed in the Supplementary Table 1. PCR products were identified by agarose gel electrophoresis.

### In vivo orthotopic tumor model and castration model

A total of 24 male 4-6 weeks old C57BL/6 mice were used. Hep 1-6-PCDH and Hep 1-6-AR cells were engineered to express the luciferase reporter gene (PCDNA3.0-luciferase) by stable transfection, and the positive clones were selected with G418 and expanded. Mice were randomly divided into 4 groups. Two groups mice were injected with Hep 1-6-PCDH cells and the two other groups were injected with Hep 1-6-AR cells with the quantity of 2 × 10^6^ of luciferase expressing cells each mouse (as a mixture with Matrigel, 1:1) into the left lobe of the liver. Tumor formation and metastasis were monitored by fluorescent imager (IVIS Spectrum). As soon as liver tumors could be detected, we started to inject the PD-L1 inhibitor (Bioxcell EB0101) intraperitoneally (i.p.) to one group of Hep 1-6-PCDH injected mice and one group of Hep 1-6-PCDH injected mice, at the dosage of 4 mg/kg, once a week. The remaining groups were injected with the IgG control (Bioxcell BE0086) at the same dosage, once a week. Mice were sacrificed after 6 weeks of injection and liver tumors were isolated for further examination.

For castration model, mice were castrated or sham-operated at 4-6 weeks of age and housed individually. All operative procedures were performed under pentobarbital anesthesia (50 mg/kg body weight, i.p. injection). Briefly, an incision was made in the wall of the abdomen. The testis with epididymis was removed following seminal duct ligation.

All animal studies were performed under the supervision and guidelines of the Sir Run Run Shaw Hospital Animal Care and Use Committee, Zhejiang University.

### Patient selection and immunohistochemistry (IHC) staining

74 formalin-fixed and paraffin-embedded HCC samples with corresponding adjacent normal tissue were selected from January 2004 to December 2010 in Sir Run Run Shaw Hospital, School of Medicine, Zhejiang University, China. All the selected patients had a 5-year follow-up. The IHC slides of all 74 patients used for AR and PD-L1 scoring were reviewed by two pathologists in a double-blind manner. The staining results were assessed semi quantitatively based on the following scale: (−), (+), (++), and (+++). The staining score was based on the following criteria: (−), less than 10% staining of nuclear AR in any of the tumor cells per field or no cytoplasmic PD-L1 staining; (+), nuclear AR staining in 10% to 30% of the tumor cells with any intensity, or faint, barely discernable cytoplasmic staining for PD-L1; (++), staining in 30% to 50% of the tumor cells with moderate-to-strong intensity of nuclear AR, or moderate, smooth cytoplasmic staining of tumor cells with moderate PD-L1 staining; (+++), staining in more than 50% of the tumor cell nuclei with strong AR staining, or apparent cytoplasm staining for PD-L1. Representative examples of (−), (+), (++), and (+++) IHC staining for AR and PD-L1 are shown in Fig 4. The antibody used for anti-PD-L1(Cell Signaling Technology 13684) was the same as used in western blot and the antibody of anti-AR (ab 198394) was purchased from Abcam.

This experiment was approved by the Institutional Review Board/Privacy Board of Sir Run-Run Shaw Hospital

### Statistical analysis

Data were expressed as the mean±SEM from at least 3 independent experiments. Statistical analyses included unpaired t-test, one-way ANOVA, and Spearman’s correlation, and were performed with SPSS 17.0 (SPSS Inc., Chicago, IL). P <0.05 was considered statistically significant. Kaplan–Meier method with log-rank test was applied to compare patients’ disease-free survival and overall survival.

## Supplementary Material

Supplementary Figures

Supplementary Table 1
