# Supplementary material for: Androgen receptor affects the response to immune checkpoint therapy by suppressing PD-L1 in hepatocellular carcinoma
Source: Aging (Albany NY). 2020 Jun 24;12(12):11466–84. doi: 10.18632/aging.103231 (PMC7343489; doi:10.18632/aging.103231)
Supplement: Supplementary Figures [file aging-12-103231-s002..pdf]

SUPPLEMENTARY FIGURES

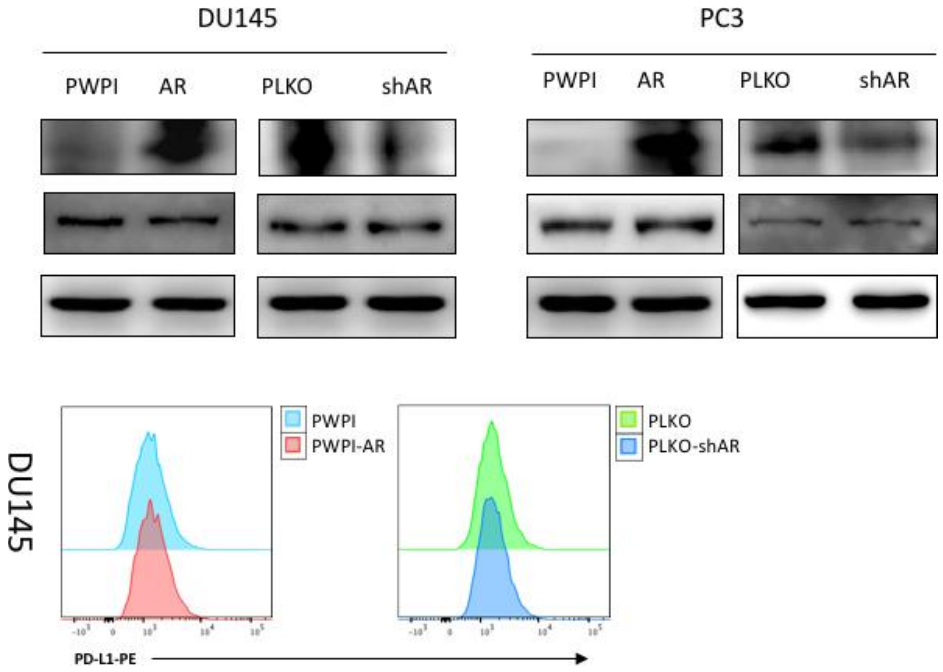

Supplementary Figure 1. Changes of PD-L1 in prostrate cell lines with difference AR expression tested by western bot and flow cytometry.

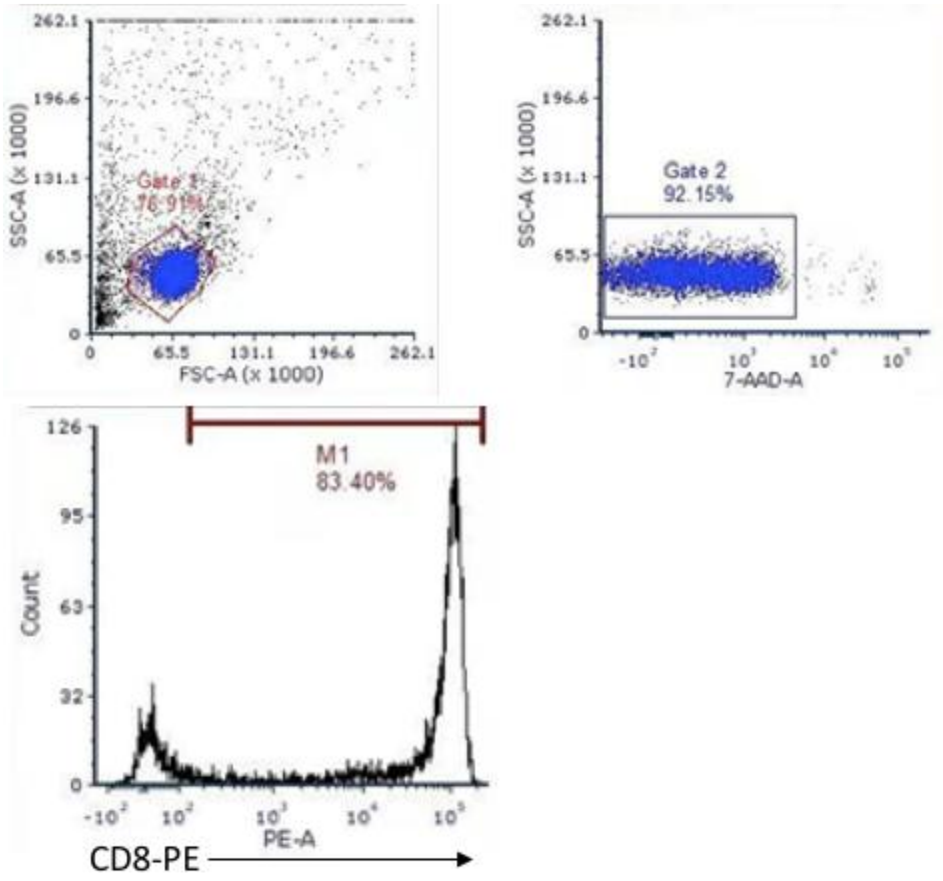

Supplementary Figure 2. The purity of extracted CD8$^+$ T cells was identified by flow cytometry. 7-AAD buffer was used to distinguish between dead and living cells and CD8$^+$ T cell was stained by CD8-PE antibody.

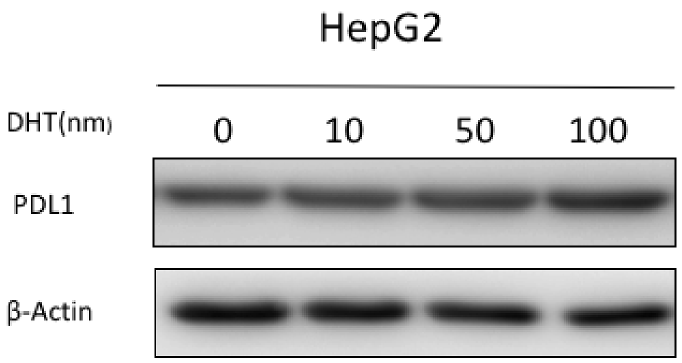

Supplementary Figure 3. The castration assay was performed in HepG2 cell. PD-L1 expression was tested by western blot.

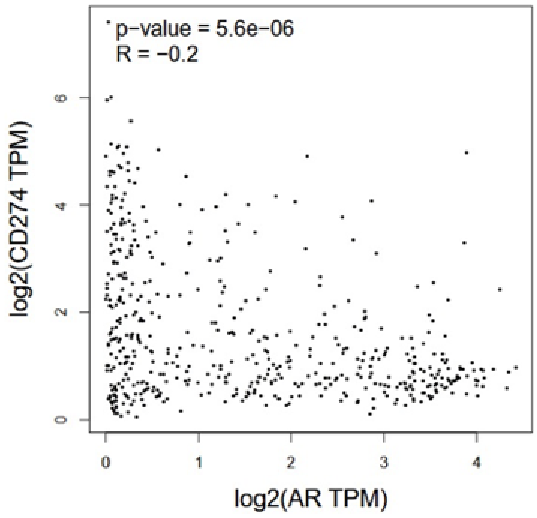

Supplementary Figure 4. Based on TCGA database, the correlation between AR and PD-Ll was verified in GEPIA (http://gepia.cancer-pku.cn/).

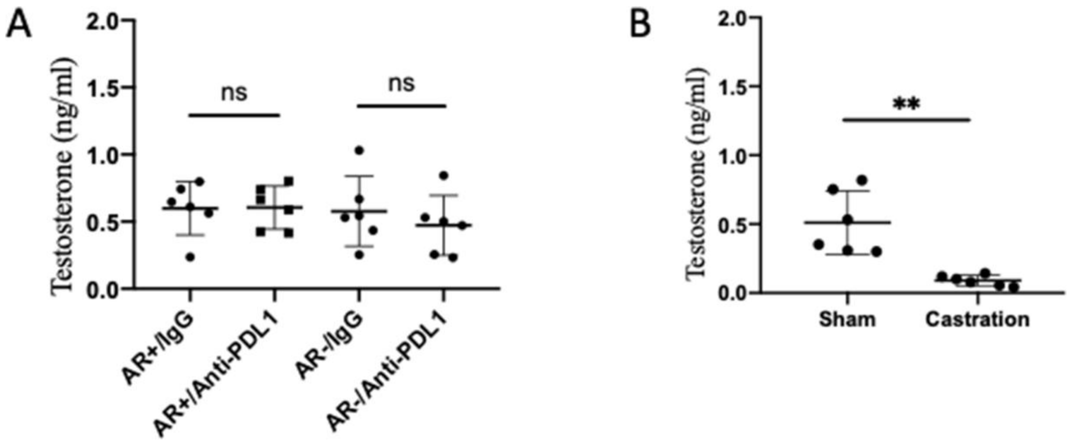

Supplementary Figure 5. The plasma androgen level of mice was measured using ELISA Kit. (**A**) The plasma androgen level between the four groups of the mice orthotopic tumor model. (**B**) The plasma androgen level of the sham-operated mice and castrated mice.
