# Supplementary material for: Androgen receptor affects the response to immune checkpoint therapy by suppressing PD-L1 in hepatocellular carcinoma
Source: Aging (Albany NY). 2020 Jun 24;12(12):11466–84. doi: 10.18632/aging.103231 (PMC7343489; doi:10.18632/aging.103231)
Supplement: Supplementary Table 1 [file aging-12-103231-s001..pdf]

SUPPLEMENTARY TABLE

Supplementary Table 1. Specific primers and promotor of CD274.

Gene	Primer	Sequence	Experiment
CD274	CD274-F	CACCGTGGCATTTGCTGAACGCATTT	RT-PCR
	CD274-R	AAACCGTCCCCCTTTCTGATAAAAGCC	
TIM3	Tim3-F	CACCGCTGCTGCTACTACTTACAAGGTC	RT-PCR
	Tim3-R	AAACGCAGGGCAGATAGGCATTCTC	
CTLA-4	CTLA-4-F	CACCGGCCCTGCACTCTCCTGTTTTT	RT-PCR
	CTLA-4-R	AAACGGTTGCCGCACAGACTTCAC	
1st ARE-1	ARE1-1-F	CACCGTGGGTCTGCTGCTGACTTTT	CHIP
	ARE1-1-R	AAACAGGCGTCCCCCTTTCTGATAC	
1st ARE-2	ARE1-2-F	CACCGAAGCCATATGGGTCTGCTGC	CHIP
	ARE1-2-R	AAACTTATCAGAAAGGCGTCCCCCC	
1st ARE-3	ARE1-3-F	CACCGTGGGTCTGCTGCTGACTTTTTA	CHIP
	ARE1-3-R	AAACCAACATCTGAACGCACCTTGATC	
2nd ARE-1	ARE2-1-F	CACCGGAGGTGGGCGGGACCCCGCC	CHIP
	ARE2-1-R	AAACCAGCGAGCTAGCCAGAGATAC	
2nd ARE-2	ARE2-2-F	CACCGGAGGTGGGCGGGACCCCGCC	CHIP
	ARE2-2-R	AAACGCCAGAGATACTGGGCCGTGC	
2nd ARE-3	ARE2-3-F	CACCGCCAGTTCTGCGCAGCTTCC	CHIP
	ARE2-3-R	AAACGCTAGCCAGAGATACTGGGCC	
ARE control	Control1-F	CACCGGTAACCTTAAGCTCTTACCC	CHIP
	Control1-R	AAACCTGTGTATAGAAATGAAACAC	

CD274 promotorTAGAAGTTCAGCGCGGGATAATACTTAAGTAAATTATGACACCATCGTCTGTCATCTTGGGCCCATTCACTAACCCAAAGCTTTCAAAAGGGCTTTCTTAACCCTCACCTAGAATAGGCTTCCGCAGCCTTAATCCTTAGGGTGGCAGAATATCAGGGACCCTGAGCATTCTTAAAAGATGTAGCTCGGGATGGGAAGTTCTTTTAATGACAAAGCAAATGAAGTTTCATTATGTCGAGGAACTTTGAGGAAGTCACAGAATCCACGATTTAAAAATATATTTCCTATTATACACCCATACACACACACACACACCTACTTTCTAGAATAAAAACCAAAGCCATATGGGTCTGCTGCTGACTTTTTATATGTTGTAGAGTTATATCAAGTTATGTCAAGATGTTCAGTCACCTTGAAGAGGCTTTTATCAGAAAGGGGGACGCCTTTCTGATAAAGGTTAAGGGGTAACCTTAAGCTCTTACCCCTCTGAAGGTAAAATCAAGGTGCGTTCAGATGTTGGCTTGTTGTAAATTTCTTTTTTTATTAATAACATACTAAATGTGGATTTGCTTTAATCTTCGAAACTCTTCCCGGTGAAAATCTCATTTACAAGAAAACTGGACTGACATGTTTCACTTTCTGTTTCATTTCTATACACAGCTTTATTCCTAGGACACCAACACTAGATACCTAAACTGAAAGCTTCCGCCGATTTCACCGAAGGTCAGGAAAGTCCAACGCCCGGCAAACTGGATTTGCTGCCTTGGGCAGAGGTGGGCGGGACCCCGCCTCCGGGCCTGGCGCAACGCTGAGCAGCTGGCGCGTCCCGCGCGGCCCCAGTTCTGCGCAGCTTCCCGAGGCTCCGCACCAGCCGCGCTTCTGTCCGCCTGCAGGTAGGGAGCGTTGTTCCTCCGCGGGTGCCCACGGCCCAGTATCTCTGGCTAGCTCGCTG
